# The Effects of Glucose Fluctuation on the Severity of Coronary Artery Disease in Type 2 Diabetes Mellitus

**DOI:** 10.1155/2013/576916

**Published:** 2013-07-07

**Authors:** Xingguang Zhang, Xiuping Xu, Xiumin Jiao, Jinxiao Wu, Shujing Zhou, Xiaofeng Lv

**Affiliations:** General Hospital of Beijing Military Region, No. 5 Nan Men Cang, Dongcheng Distrct, Beijing 100700, China

## Abstract

*Objectives*. To explore the difference of glucose fluctuations between the normal subjects and diabetes mellitus (DM) patients and explore their impact on the development of CAD in type 2 DM patients. *Methods*. The subjects were divided into 3 groups: normal control (group A, *n* = 40), type 2 DM patients without cardiovascular complications (group B, *n* = 56), and type 2 DM patients with cardiovascular complications (group C, *n* = 92). The SYNTAX scores were collected in group C. CGMS for 72 h was applied on all the subjects. The indexes such as MBG and the LAGE were calculated. Glycemic excursions were compared between groups A, B, and C, respectively. *Results*. The tested indexes had significant differences among the three groups. SYNTAX scores are related to systolic blood pressure, CRP, MAGE, and HbA1c and are significantly correlated at 6:00–8:00 and 11:00–13:00 time points in group C. *Conclusions*. Compared with normal subjects, T2DM patients have greater blood glucose fluctuations; T2DM patients with CAD have larger glucose fluctuations than T2DM patients without CAD. Blood glucose fluctuations are positively correlated with carotid artery intima-media thickness in T2DM patients and have a significant influence on the development of coronary artery.

## 1. Introduction

The incidence of type 2 diabetes mellitus (T2DM) is increasing these years with the improvement of people's living standard, the changes of life style, and the increasing aging population. Yang et al. reported that the prevalence of diabetes in adults over 20 years old was 9.7% and the prevalence of prediabetes (impaired fasting glycaemia and impaired glucose tolerance) has reached 15.5% [1]. The complications of T2DM almost involved each organ of the body; 60%–80% of the patients died of vascular disease [2]. Large vascular disease affects the aorta, coronary artery, cerebral artery, renal artery, and peripheral artery mainly, which is hard to ignore; many researchers have studied the effect of blood glucose fluctuation on the vascular complications of T2DM [3–6]. Quagliaro et al. confirmed that the blood vessel endothelium was damaged greater by blood glucose fluctuation than by chronic persistent hyperglycemia [6]; recent studies have demonstrated that acute and chronic fluctuations in blood glucose levels can increase oxidative stress in type 2 diabetes mellitus patients [7], which results in cell dysfunction and tissue injury [8]. Therefore, it is important to evaluate the relationship between the blood glucose fluctuation and the coronary artery disease by dynamic glucose monitoring. Su et al. [9] have reported that the intraday glycemic variability is associated with the presence and severity of CAD in patients with T2DM, and effects of glycemic excursions on vascular complications should not be neglected in diabetes. However, the significance and value of this study were limited by small population and less correlation analysis of some important medical indexes, such as mean blood glucose (MBG), glucose standard deviation (SD), the largest amplitude of glycemic excursions (LAGE), the average amplitude of glycemic excursions (MAGE), the number of effective blood glucose excursions (NEGE), and postprandial glucose excursions of 3 dinners (PPGE1, PPGE2, and PPGE3). 

Aiming to evaluate the coronary artery disease, coronary angiography and new complexity of coronary artery disease scoring method (SYNTAX scores) were used in the current study [10]. Coronary angiography is accepted as a golden standard for the diagnosis of coronary heart disease. The SYNTAX score is a complete angiography scoring system and can be used for the comprehensive evaluation of coronary lesion. The higher the score is, the more severe it may be; following treatment may be more difficult and the prognosis may be worse [11].

In this study, we explored the effect of chronic blood glucose fluctuation on coronary artery disease through studying T2DM patients with or without cardiovascular complications. Compared to previous similar studies [9], SYNTAX score system was used to estimate the severity of coronary lesion through coronary angiography findings, which is significant for the diagnosis of large vascular complications in patients with T2DM. We also provided more adequate indexes, such as MBG, SD, LAGE, MAGE, NEGE, PPGE1, PPGE2, and PPGE3. We also analyzed the correlation of HOMA-IR, HOMA-*β* function index (HBCI), and IMT with glucose fluctuations.

## 2. Patients and Methods

### 2.1. Subjects

The study has been approved and registered by our hospital's Ethics Committee in January 2012; the Ethics Committee approved related screening, treatment, and data collection of these patients; written informed consent was obtained from each patient for the use of their blood sample and clinical information. All works were undertaken following the provisions of the Declaration of Helsinki.

The inclusion criteria of DM patients were 50 to 69 years old; gender not limited; type two diabetes mellitus diagnosed according to the WHO diagnostic criteria in 1999 [12], admission glucose <16.7 mmol/L, and without diabetic ketosis or nonketotic hyperosmolar coma; cardiovascular risk equal or higher than 10% according to continuous metabolic syndrome risk score [13]. They were excluded if they have known coronary artery disease; symptomatic heart failure; objective inability to perform treadmill exercise; known or active malignancy, advanced renal failure (serum creatine clearance <25 mL/mi/1.73 m^2^), and liver cirrhosis (child Pugh III); stroke within the past 30 days; presence of left bundle branch block or ST depression at rest greater than 0.9 mm.

A total of 148 patients diagnosed with T2DM were enrolled in this study; these patients were suspected with coronary artery disease. The coronary angiography was conducted to get the information of their coronary lesion in General Hospital of Beijing Military Region, PLA. Healthy individuals without history of diabetes mellitus and coronary artery disease were recruited from volunteers of community inhabitants in Beijing (matched with age range); they were set as normal control group (group A, *n* = 40).

## 3. Coronary Angiography

Coronary artery angiography was performed by using standard Judkins techniques or a radical approach. During cardiac catheterization, nitroglycerine was administrated routinely in all cases suspected of having coronary spasm. Angiographic analysis was carried out by two experienced interventional cardiologists who were blinded to the study protocol. Angiography results were divided into CAD (≥50% obstruction in ≥1 coronary artery) group and non-CAD group. According to coronary angiography results, 148 patients were divided into 2 groups: patients without cardiovascular complications (group B, *n* = 56) and with cardiovascular complications (group C, *n* = 92). 

## 4. Routine and Biochemical Examinations

General clinical data such as the gender, age, body mass index (BMI), antihypertension drug, systolic pressure (SBP), and diastolic pressure (DBP) were recorded by routine medical examination.

Blood samples were obtained under overnight fasting conditions from these patients, and high-density lipoprotein cholesterol (HDL-c), low-density lipoprotein cholesterol (LDL-c), triglyceride (TG), total cholesterol (TC), and C reactive protein (CRP) of all subjects were measured by routine blood examination. Serum concentration of hemoglobin A1c (HbA1c) was determined by high-performance liquid chromatographic method using automatic HbA1c analyzer (Tosoh HLC-723G7, Japan). The SYNTAX scores in group C were calculated with the help of professional website tool: http://www.syntaxscore.com/. 

Meanwhile, we tested the plasma-reduced glutathione (GSH) level in each group to evaluate the oxidative stress; GSH was determined with a colorimetric assay using Bioxytech GSH-400 kit (Oxis International, Portland, OR, USA) based on a two-step reaction: thioethers formation followed by a *β*-elimination under alkaline conditions. Thioethers obtained are transformed into chromophoric thiones, which have a maximal absorbance wavelength at 400 nm. 

### 4.1. Dynamic Blood Glucose Monitoring

The HOMA-IR and HBCI were calculated with the homeostasis model assessment. HOMA-IR = fasting blood glucose (FBG) × fasting insulin (FINS)/22.5, HBCI = 20 × FINS/(FBG-3.5) [14].

Continuous glucose monitoring system (CGMS) for 72 h was applied for all the subjects. A CGMS sensor was inserted into the subcutaneous abdominal fat tissue, calibrated according to the standard Medtronic MiniMed operating guidelines. The indexes of mean blood glucose (MBG), glucose standard deviation (SD), the largest amplitude of glycemic excursions (LAGE), the average amplitude of glycemic excursions (MAGE), the number of effective blood glucose excursions (NEGE), and postprandial glucose excursions of 3 dinners (PPGE1, PPGE2, and PPGE3) were recorded separately by CGMS. 

The MAGE was calculated by measuring the arithmetic mean of the differences between consecutive peaks and nadirs, provided that the differences are greater than one standard deviation of the mean glucose value. The MODD was calculated as the mean of the absolute differences between glucose values at the same time of two consecutive days. The PPGE was obtained by calculating the postbreakfast increments in blood glucose.

Glycemic excursions were compared between groups A, B, and C. The key factors impacting SYNTAX scores were analyzed in group C by multiple linear regression analysis. Blood glucose fluctuation was recorded at 8 time sessions, which were 0:00–3:00, 3:00–6:00, 6:00–8:00, 8:00–11:00, 11:00–13:00, 13:00–17:00, 17:00–19:00, and 19:00–24:00; the correlations with SYNTAX scores were analyzed in every section.

### 4.2. Statistical Analysis

If the data were normal distribution data, *t*-test was used in comparison of two groups and single factor analysis of variance was used in comparison of three groups. If the data were nonnormal distribution data, rank-sum test was used in comparison between two groups and the Kruskal-Wallis analysis was used in comparison of three groups. The Chi-square test was used for qualitative data test; multiple factors were analyzed by multiple linear regression analysis. All analyses were performed using SPSS software program, version 16.0, for Windows (SPSS Institute Inc.) and *P* < 0.05 was considered statistically significant.

## 5. Results

### 5.1. The Comparison of the Indexes among the Three Groups

After coronary angiography, DM patients were divided into group B and group C.  Table 1 showed the detailed data of the three groups. Compared with group A (healthy control), patients from group B have significantly higher BMI (27.0 ± 3.6 versus 23.8 ± 2.5), antihypertension drug using rate (55.4% versus 10%), HbA1c (6.6 ± 1.2 versus 5.3 ± 0.3%), HDL-c (0.96 versus 0.88 mmol/L), MBG (8.1 ± 2.1 versus 6.1 ± 0.6 mmol/L), MAGE (2.6 versus 2.2 mmol/L), SDBG (1.5 ± 0.4 versus 0.8 ± 0.3 mmol/L), LAGE (6.6  versus  2.9 mmol/L), IMT (0.9 ± 0.3 versus 0.6 ± 0.1 mm), and CRP (1.1 versus1.0 mg/L) and significantly lower NEGE (4.1 versus 6.8 times), HBCI (45 versus 87), and GSH level (3.18 ± 0.86 versus 4.23 ± 0.64 mmol/L); *P* < 0.05 or 0.01.

Consistent with group B, as shown in Table 1, and compared with group A, group C had the same trend with significantly higher age, antihypertension drug using rate, SBP, DBP, HbA1c, MBG, MAGE, SDBG, LAGE, PPGE1, PPGE 2, PPGE3, IMT, CRP, HOMA-IR, and GSH and lower NEGE value (*P* < 0.05 or *P* < 0.01).

Compared with group B patients, group C patients have significantly higher age (61.7 ± 7.2 versus 56.1 ± 6.6 yr), SBP (136 ± 18 versus 124 ± 9 mmHg), DBP (80 versus 72 mmHg), MAGE (4.0 versus 2.6 mmol/L), SDBG (2.0 ± 0.8 versus 1.5 ± 0.4 mmol/L), PPGE3 (4.7 ± 2.5 versus 2.7 ± 1.1 mmol/L), IMT (1.1 ± 0.3 versus 0.9 ± 0.3 mm), CRP (3.8 versus 1.1 mg/L), and HBCI (80 versus 45) and lower NEGE (4.0 versus 6.8 times) and GSH level (2.24 ± 0.73 versus 4.23 ± 0.64 mmmol/L). *P* < 0.05 or *P* < 0.01. 

### 5.2. The Linear Correlation Analysis between SYNTAX Scores and Relative Factors in Group C

Multiple linear regression analysis showed the SYNTAX scores were significantly correlated with CRP, MAGE, and HbA1c in group C (*P* < 0.05) and were significantly correlated with SBP (*P* < 0.01) (Table 2).

### 5.3. The Correlation Analysis between SYNTAX Scores and the Blood Glucose Excursion of Different Time Sessions in Group C

In our study, a day was divided into eight sessions. Significant correlations were found in 6:00–8:00 (*P* < 0.01) and 11:00–13:00 (*P* < 0.05) between the SYNTAX scores and blood glucose excursion in group C (Table 3). 

### 5.4. The Linear Correlation Analysis between MAGE and the Related Factors in Groups B and C

After analysis, MAGE was positively correlated with age, CRP, HbA1c, HOMA-IR, IMT, SD, MBG, LAGE, and glucose excursions before and after meals (*P* < 0.01) and was negatively correlated with HBCI (*P* < 0.05) and NEGE (*P* < 0.01) both in groups B and C (Table 4). 

## 6. Discussion

The risk of coronary heart disease (CHD) mortality in type 2 diabetic patients is more than twofold higher compared with that in age-matched healthy subjects. The incidence of stroke events and all manifestations of CHD, myocardial infarction (MI), sudden death, and angina pectoris are at least twofold higher in patients with type 2 diabetes than in nondiabetic individuals [15]. Therefore, it is important to investigate the relevant affecting factors which cause such high risk.

Brownlee found that too much mitochondrial reactive oxygen species (ROS) may be a common mechanism of diabetic complications [16]. The oxidative stress was enhanced by the blood sugar disorder in T2DM patients; the uncompensated antioxidant capacity in vivo leads to endothelial damage, thus causing macrovascular complications. In the current study, the lowest CGH level in group C also proved this point. CRP, a biomarker of cardiovascular diseases [17], was highly related to MAGE in our study. Glucose excursions in subjects with impaired glucose regulation and T2DM trigger the activation of oxidative stress [18]; MAGE was correlated with HOMA-IR positively and negatively correlated with HBCI, which suggests MAGE could affect the insulin resistance and the function of pancreatic islets. 

Recently, large-scale clinical studies have suggested that only using HbA1c for strict glycemic control is not sufficient to reduce the risk of macrovascular complications [19, 20]. The effects of blood glucose fluctuation on vascular complications in T2DM have been researched by many scientists. Hanefeld M. et al. found that postprandial blood glucose peak can predict myocardial infarction better than fasting glucose [21]; Ceriello et al. demonstrated that accelerated oxidative stress accompanying fluctuations in blood glucose levels could worsen endothelial dysfunction more than constant hyperglycemia [22]; Torimoto et al. found that fluctuations in blood glucose levels play a significant role in vascular endothelial dysfunction in T2DM [23]; Su et al. found that the glucose variability was closely associated with the severity of cardiovascular disease in T2DM; the effect of MAGE on coronary artery was greater than that of HbA1c [9]; Colette and Monnier suggested that the MAGE can serve as the gold standard to measure the blood glucose fluctuation [24]. We found that MAGE in T2DM patients with coronary artery disease was higher than that in T2DM patients without coronary artery disease. Multiple linear regression analysis suggested that both HbAlc and MAGE were important factors affecting the cardiovascular complications of T2DM, but MAGE was more predictive than HbAlc. This study also showed that there was significant correlation between MAGE and IMT. IMT can be used as the index of early atherosclerosis.

In this study, MAGE was positively correlated with age, CRP, HbA1c, HOMA-IR, IMT, SD, MBG, LAGE, and glucose excursions before and after meal and negatively correlated with HBCI and NEGE. It suggested that MAGE may be influenced by the above factors. 

The shortage of this research lies in that the impact of other factors such as blood pressure, age, and HbA1c cannot be completely ruled out although the multiple linear regression analysis has been used; these previous factors also can influence progression of CAD. Group C patients have higher age and antihypertension drug using rate; 85% of the blood pressure of them is below or near the critical range 140/90 mm Hg. Previous studies have reported that DM patients with pressure below 140 mm Hg can benefit from aggressive antihypertensive treatment [25]. Besides that, other few limitations of this study should be mentioned. Firstly, the sample size was relatively small in this study, so some subgroup comparisons may have lacked power to detect significant differences for selected variables. Secondly, although we had maintained the patients antihyperglycemic therapy as usual and avoided glucose infusion during CGMS monitoring period, some factors, such as different diets and physical and emotional stress, which may affect levels of admission glucose fluctuations could not be all prevented. Thirdly, lack of microvascular complications data in another limitation; we did not include those risk factors in study. 

Currently, although there is still an extensive debate about glucose fluctuation as a risk factor for complications independent of HbA1c in diabetes [26, 27], by this present study, we provide some evidence to suggest that, at least, glucose fluctuation has potential to be a risk factor for predicting the occurrence and progression of CAD; it can be helpful to test this index in clinical treatment for DM patients. 

## 7. Conclusions

Compared with normal subjects, T2DM patients have greater blood glucose fluctuations and higher average blood glucose. T2DM patients with larger glucose fluctuations could have higher risk for coronary artery disease compared with patients having smaller glucose fluctuations. Compared with high blood glucose, blood glucose fluctuations may be more importantly influential on the development of coronary artery disease in patients with T2DM. Blood glucose fluctuation is significantly related to carotid artery intima-media thickness in T2DM.

## Figures and Tables

**Table 1:** Comparison of the indexes in the three groups.

	Group A	Group B	Group C
Sex (M/F)	24/16	24/32	48/44
Age (years)	56.3 ± 6.1	56.1 ± 6.6	61.7 ± 7.2^∗#^
BMI (Kg/m^2^)	23.8 ± 2.5	27.0 ± 3.6*	24.5 ± 1.7
CD (year)	—	6.10 (4.54, 8.11)	4.73 (2.77, 7.70)
Antihypertension drug user (%)	4 (10%)	31 (55.4%)**	55 (59.8%)**
SBP (mmHg)	114 ± 13	124 ± 9	136 ± 18^∗∗#^
DBP (mmHg)	70 (65–75)	72 (66–79)	80 (80-81)^∗#^
HbA1c (%)	5.3 ± 0.3	6.6 ± 1.2**	7.5 ± 1.4^∗∗#^
HDL-c (mmol/L)	0.88 (0.83–0.92)	0.96 (0.92–1.27)*	0.91 (0.85–1.13)
LDL-c (mmol/L)	2.16 (2.05–2.20)	2.51 (1.78–2.92)	2.17 (2.07–3.23)
TG (mmol/L)	1.63 (1.58–1.66)	2.12 (1.21–4.11)	1.70 (1.42–2.23)
TC (mmol/L)	4.14 (3.93–4.25)	4.74 (4.05–4.79)	4.11 (3.57–4.46)
GSH (mmol/L)	4.23 ± 0.64	3.18 ± 0.86	2.24 ± 0.73
MBG (mmol/L)	6.1 ± 0.6	8.1 ± 2.1*	8.0 ± 1.4**
MAGE (mmol/L)	2.2 (1.8, 2.8)	2.6 (1.9, 3.5)**	4.0 (3.3, 4.8)^∗∗##^
NEGE (times)	6.8 (6.0, 7.7)	4.1 (3.5, 4.7)**	4.0 (3.4, 4.8)**
SDBG (mmol/L)	0.8 ± 0.3	1.5 ± 0.4**	2.0 ± 0.8^∗∗#^
LAGE (mmol/L)	2.9 (2.6–4.5)	6.6 (5.8–12.4)**	7.8 (6.1–9.9)**
PPGE1 (mmol/L)	2.7 (1.6–4.3)	3.2 (1.7–9.8)	4.6 (3.2–8.1)**
PPGE2 (mmol/L)	2.3 (1.7–3.5)	2.4 (2.2–8.6)	3.3 (2.6–5.4)*
PPGE3 (mmol/L)	2.2 ± 0.4	2.7 ± 1.1	4.7 ± 2.5^∗∗##^
IMT (mm)	0.6 ± 0.1	0.9 ± 0.3*	1.1 ± 0.3^∗∗#^
CRP (mg/L)	1.0 (0.9–1.1)	1.1 (1.0–2.8)*	3.8 (2.8–5.4)^∗∗##^
HOMA-IR	1.58 (1.52–1.69)	2.3 (1.9–9.1)**	4.3 (3.0–4.9)**
HBCI	87 (82–103)	45 (28–67)**	80 (48–139)^##^
SYNTAX score	—	0	23.5 ± 5.9

CD: course of disease. Compared with group A, *P < 0.05, **P < 0.01; compared with group B, ^#^ *P* < 0.05, ^##^ *P* < 0.01.

**Table 2:** Linear correlation analysis between SYNTAX scores and relative factors in group C.

SYNTAX scores	Age^*▼*^ (years)	BMI^*▼*^ (Kg/m^2^)	SBP^*▼*^ (mmHg)	DBP^▲^ (mmHg)	CD^*▼*^ (year)	CRP^*▼*^ (mg/L)	HbA1c^*▼*^ (%)
*R*	−0.115	0.046	0.551	−0.015	−0.298	0.435	0.488
*P*	0.602	0.836	0.006	0.947	0.167	0.038	0.018

SYNTAX scores	MAGE^*▼*^ (mmol/L)	HOMA-IR^▲^	HBCI^▲^	HDL-c^▲^ (mmol/L)	LDL-c^▲^ (mmol/L)	TC^▲^ (mmol/L)	TG^▲^ (mmol/L)

*R*	0.518	−0.199	−0.040	0.329	0.183	−0.069	−0.059
*P*	0.011	0.363	0.855	0.125	0.403	0.754	0.789

CD: course of disease. ^*▼*^Pearson's correlation analysis. ^▲^Spearman's correlation analysis.

**Table 3:** Correlation analysis between SYNTAX scores and the blood glucose excursion of different time sessions in group C.

SYNTAX scores	0:00–3:00 (mmol/L)	3:00–6:00 (mmol/L)	6:00–8:00 (mmol/L)	8:00–11:00 (mmol/L)
*R*	−0.442	−0.208	0.678	0.115
*P*	0.035	0.340	0.000	0.600

SYNTAX scores	11:00–13:00 (mmol/L)	13:00–17:00 (mmol/L)	17:00–19:00 (mmol/L)	19:00–24:00 (mmol/L)

*R*	0.523	0.257	0.358	−0.018
*P*	0.011	0.237	0.094	0.933

Pearson correlation analysis was used in 0:00–3:00 and Spearman's correlation analysis was used in other's time sessions.

**Table 4:** Linear correlation analysis between MAGE and the related factors in groups B and C.

MAGE	Age	BMI	CD	CRP	HbA1c	HOMA-IR	HBCI	IMT
	(years)	(Kg/m^2^)	(year)	(mg/L)	(%)			(mm)
*R*	0.383	−0.193	−0.065	0.599	0.595	0.498	−0.297	0.460
*P*	0.008	0.193	0.702	0.000	0.000	0.000	0.042	0.001

MAGE	NEGE	SD	MBG	LAGE		PPGE1	PPGE2	PPGE3
	(time)	(mmol/L)	(mmol/L)	(mmol/L)		(mmol/L)	(mmol/L)	(mmol/L)

*R*	−0.712	0.928	0.576	0.862		0.764	0.631	0.672
*P*	0.000	0.000	0.000	0.000		0.000	0.000	0.000

CD: course of disease. All are analyzed with Spearman's correlation analysis.

## Abbreviations

BMI: Body mass index
CD: Course of disease
CGMS: Continuous glucose monitoring system
CRP: C reactive protein
DBP: Diastolic pressure
DM: Diabetes mellitus
HbA1c: Hemoglobin A1c
HBCI: HOMA *β*-cell function index
HDL-c: High-density lipoprotein cholesterol
HOMA-IR: HOMA insulin resistance index
LAGE: Largest amplitude of glycemic excursions
LDL-c: Low-density lipoprotein cholesterol
MAGE: Mean amplitude of glycemic excursions
MBG: Mean blood glucose
NEGE: Number of effective glucose excursions
PPGE1: Postprandial glucose excursions of breakfast
PPGE2: Postprandial glucose excursions of lunch
PPGE3: Postprandial glucose excursions of supper
SBP: Systolic pressure
SDBG: Standard deviation of blood glucose
TC: Total cholesterol
TG: Triglyceride
SYNTAX: Synergy between PCI with taxus and cardiac surgery
CAD: Coronary artery disease.
